# Changes in discourse on unmet need for family planning among married women in India: evidence from NFHS-5 (2019–2021)

**DOI:** 10.1038/s41598-023-47191-9

**Published:** 2023-11-22

**Authors:** S. K. Singh, G. C. Kashyap, Himani Sharma, Sudipta Mondal, C. H. Legare

**Affiliations:** 1 Department of Survey Research and Data Analytics, International Institute for Population Sciences, Deonar, Mumbai, 400088 India; 2 Institute of Health Management Research, Bangalore, 560105 India; 3 Measurement, Learning and Evaluation, Project Concern International (PCI), New Delhi, 110020 India; 4 Department of Psychology, Center for Applied Cognitive Science, Population Research Center, The University of Texas at Austin, Austin, USA

**Keywords:** Health care, Health policy, Health services, Public health

## Abstract

Unmet needs for contraception in India have declined over time but the rate has not been uniform among women across geographies and socio-economic strata. Identifying the characteristics of women in communities where unmet need is still high is important to devise appropriate strategies to ensure access and uptake of modern contraceptive methods. The current study examined whether there was a national decline in unmet need over time and if regional disparities exist in unmet need. Demographic variations in unmet need based on factors such as maternal age, education, religion, caste, wealth index quintile, family size, and access to antenatal care (ANC) were also documented. Our approach was to document the prevalence of total unmet need for family planning and unmet need for spacing among married Indian women and quantify variability based on socio-economic and demographic drivers within a hierarchal framework, thus providing both macro and micro perspectives. We used data from the fourth and fifth rounds of the National Family Health Survey (NFHS) collected from all the States and Union Territories (UTs) in India. Quantile regression analysis and multilevel regression techniques were used to understand the predictors for the total unmet need for family planning and the unmet need for spacing. Results show a considerable decline in the prevalence of unmet need for family planning in India from NFHS-4 to 5 (from 12.9 to 9.3%) in the last six6 years. The north-eastern states show a significant reduction in unmet need for family planning in Manipur (17.8%), Nagaland (13.5%), and followed by Sikkim (9.1%). The predictors such as years of schooling, place of residence, caste, religion, wealth quintile, number of antenatal care (ANC) visits, and children ever born have a significant association with unmet needs for family planning and spacing among married women in India. There is a significant association between years of schooling with the total unmet needs for family planning at (q25) quantiles and the unmet need for spacing at (q25, q50) quantiles. Results reveal that the demand for unmet need for spacing and limiting was the highest among the women in the age categories 15–19 (17.8%) and 20–24 (17.3%). The demand for limiting was the highest (6.8%) among Muslim women. Across wealth quantile categories, the overall unmet demand (11.4%) for spacing and limiting was the highest among the women in the lowest socioeconomic groups. We conclude that greater access to frontline health workers among young wives, and significant investment in education in general, will continue to reduce the unmet needs for family planning in India.

## Introduction

At the International Conference for Population and Development (ICPD) in 1994, the global community of public health leaders, experts, policymakers, and implementers advocated for family planning to support women and girls’ right to decide freely and for themselves whether, when, and how many children they want to have^1^. The summit's goal was to reach 120 million additional users of modern contraceptive methods in the world's poorest countries by 2020^2^. In India, this program has been implemented by the Ministry of Health and Family Welfare (MoHFW). MoHFW had committed to covering additional 48 million women with modern contraceptives by 2020^3^.

An unmet need for contraception occurs when women are married or living in a sexually active union, do not use any contraception method, and do not want to have more children^4^. The unmet need for contraception portrays a clear picture of the differences between women's reproductive intention and contraceptive practices. The goal in meeting the contraceptive need for women or couples is to prevent unwanted, closely spaced, or poorly timed pregnancies, all of which affect maternal and infant morbidity and mortality^5^. There are two types of unmet needs—unmet needs for spacing and unmet needs for limiting. The sum of these two types of unmet needs provide the estimate for overall unmet needs. Both the types of unmet needs need to be examined separately as they have a significant programmatic implication. Unmet needs for spacing is more relevant to younger women, while changing unmet needs for limiting needs more focus on women with a higher parity and age.

Unmet need for contraception have declined over time in India. The unmet need for contraception among women between the reproductive ages (15–49) dropped from over 23 percent in NFHS-3^6^ to 12.9 percent in NFHS-4^7^. Notably, the rate of declining unmet need has not been even across states and districts^8^. As per NFHS-5, the unmet needs for spacing and limiting in India were 4% and 5.4%, respectively, whereas the unmet demand for family planning was 9.4%. Notably, 87.9% of the demand for contraception was satisfied. Identifying the characteristics of women in communities where unmet need is still high is important to devise appropriate strategies to ensure access and uptake of modern contraceptive methods. The government of India introduced a program in 2016, "Mission Parivar Vikas," under the Nation Health Mission to improve access to contraception and family planning services in 145 high-fertility districts across seven empowered action group (EAG) states where the total fertility rate was three children or above. These high-needs districts are located in the seven states of Uttar Pradesh, Bihar, Madhya Pradesh, Rajasthan, Jharkhand, Chhattisgarh, and Assam, which account for 44% of the nation's population and have the highest total fertility rates of three or more^9^. A study was conducted by the United Nations Population Fund (UNFPA), to calculate the program's impact. The results show that, as compared to non-MPV districts, the districts participating in the Mission Parivar Vikas (MPV) program had lower levels of family planning outcomes before the program. The use of modern contraceptives increased more quickly in the program districts during implementation, demonstrating the effectiveness of MPV interventions^10^. The efficacy of this program provides evidence that family planning practices can be improved through government interventions.

Global estimates indicate that the unmet need for contraception is the highest among women below 20 years and the lowest among 35 years and older; these differences between age gaps have expanded in south-central Asia, including India^11^. In the Indian context, many marry young; thus, promoting contraception to postpone the first pregnancy is critical^12^. There are many different methods and strategies for supporting family planning, however, reducing the fertility rate via female sterilization has been emphasized more than delaying births. Delaying births is more relevant to young women building a family, especially those aged 20–24 and married before the legal age^13^.

Previous studies reveal that nearly one in five women in India expressed an unmet need for family planning and has identified several pertinent socioeconomic and demographic predictors across the individual, community, and district levels^14–16^**.** Across wealth quantile categories, the overall unmet demand (11.4%) for spacing and limiting was the highest among the women belonging to the lower strata of society. A study conducted in Nepal also reported similar predictors for unmet needs for family planning, such as women's current age, number of living children, education level, caste/ethnic affiliation, and residence^17^.

Without identifying the predictors that give rise to unmet needs among the young women, the family planning program will continue to witness insufficient spacing between marriage and first conception among young women. To systematically record the evolving trends in unmet needs and pinpoint the precise determinants of these needs among women residing in high needs regions we examined three questions: (1) Has unmet need declined nationally? (2) Is there regional variation in unmet need? (3) Is there a demographic variation in unmet need based on maternal age, education, religion, caste, wealth index, family size, and access to antenatal care (ANC)?

## Material and methods

### Data source

The study utilized the data from the fourth and fifth rounds of the National Family Health Survey (NFHS). The NFHS-4 and NFHS-5 surveys were conducted during 2015–2016 and 2019–2021, respectively.

NFHS survey provides extensive data on health and family welfare and emerging issues in these areas. The NFHS-5 sample was designed to provide national, state/UT, and district-level estimates of various indicators critical to monitoring the SDGs on population, health, nutrition, and gender equality, among others. However, indicators like sexual behavior; women's work, HIV/AIDS knowledge, attitudes, and practices; domestic violence; and men's health are provided only at the state/union territory (UT) and national levels. In addition, NFHS-5 has been expanded to include new issues such as the extent of preschool education, disability, access to a toilet facility, death registration, bathing practices during menstruation, and methods and reasons for abortion. The scope of clinical, anthropometric, and biochemical (CAB) testing has been expanded to include measurement of waist and hip circumferences and malaria testing^18^. The last two rounds of the survey used a similar design, and the definitions of the common indicators remained the same. It allows for easy comparison between data from the last two rounds with minimal issues with measurement validity.

### Sampling design and survey instruments

The sampling design of NFHS-5 was developed with NFHS-4 as the benchmark, and the need to provide estimates of population, health, and family welfare indicators at district, state/UT, and national levels with reasonable precision. A stratified two-stage sampling design was adopted in rural and urban areas of the 707 districts (as of Mar 31, 2017). Within each rural stratum, villages were selected from the sampling frame using probability proportional to size (PPS) with explicit stratification based on the percentage of SC/ST population and female literacy. NFHS-5 covered 609,120 households with eligible women aged 15–49 and eligible men aged 15–54 from a subsample of PSUs/households in 30,456 primary sampling units (PSU) comprised of villages in rural areas and census enumeration blocks (CEBs) in urban areas. The selection of households was based on the sampling frame prepared from mapping and listing households in all PSUs identified across 707 districts. NFHS data contains information about women 15–49 years who were asked questions about their socio-demographic characteristics, contraceptive use, reproductive histories, unmet need for family planning (spacing and limiting), future intention to use contraception, and their fertility preference. NFHS uses four types of survey instruments covering a range of issues and information on health and wellbeing. There are multiple questionnaires: the general household, male and female head of household, clinical, anthropometric, and biomedical (CAB). The CAB questionnaire contains information and records on biological markers primarily collected using standard tests in the community-based field survey.

The information analyzed in this paper was primarily collected using household and woman's questionnaires. The sample size in NFHS-4 and NFHS-5 are decided based on scientific principles to provide valid and reliable national, state, and district estimates. The overall sample size in NFHS-4 was 601,509 households and 699,686 women aged 15–49. The corresponding sample size for NFHS-5 was 636,699 Households and 724,115 women aged 15–49. Information on contraceptive use vis-a-vis met or unmet contraception needs were collected only from married women. Thus, the final sample size includes 117,481 women—68,626 from NFHS-4 and 48,855 from NFHS-5. These women reported that they did not want to have any more children in the future or at least for the next 2 years but do not use any method of contraception. We have thoroughly explained the selection process of desired sample size of women through a decision tree table in the supplementary file (Table S2).

### Variable description

#### Outcome variable

We considered two dependent variables in the study: total unmet need for family planning and unmet need for spacing for currently married women aged 15–49 (coded as 0 ‘no unmet need of contraception’, and 1 ‘If the subject reported unmet need’). We use the definition of unmet needs for family planning from the NFHS-4 survey. In the estimation of unmet need for family planning, the denominator included *all women, currently married women, and sexually active unmarried women aged 15–49. The definition computed the following aspects percentage of women who are not pregnant and not postpartum amenorrhoeic, are considered fecund and want to postpone their next birth for 2 or more years or stop childbearing altogether, but are not using any contraception method, or have a mistimed or unwanted current pregnancy, or are postpartum amenorrhoeic and their last birth in the last 2 years was mistimed, or unwanted was considered as the numerator in the estimation of unmet need for family planning. Infecund women were not included in the unmet need for family planning estimation*^7^. The unmet need for family planning consists of the unmet need for spacing and the unmet need for limiting. In the Indian scenario, most couples use permanent contraception methods like sterilization to limit births, while in the case of spacing the births, couples have to use temporary contraception more frequently. Therefore, we have considered the total unmet need for family planning and the unmet need for spacing births in the study.

#### Independent variables

A set of socio-demographic factors affecting the unmet need among currently married aged 15–49 were identified from the existing literature and used in the present study. Socioeconomic and demographic factors like age (15–19, 20–24, 25–29, 30–34, 35–39, 40–44, 45–49), Residence (Urban, Rural), Religion (Hindu, Muslim, Christian, Sikh, Buddhist/Neo-Buddhist, Jain, Other), Caste/tribe (Scheduled caste, scheduled tribe, Other backward class, Other, don’t know), Wealth quintile (Lowest, Second, Middle, Fourth, Highest), Sanitation Facility (yes/no), schooling (years of schooling), Child Marriage (yes/no), Birth Order (number of births), FP worker talked to a non-user (yes/no), ANC visits (number of ANC visits), Skilled birth attendant (yes/no), Urban Population (yes/no), SC/ST Population (yes/no), Female Literacy (yes/no) were taken in the analysis. In the multilevel analysis, factors like age (15–29, 30–39, 40–49), Schooling (No schooling, up to 5 years, < 5 to 10 years, < 10 years), Religion (Hindu, Muslim, others), Caste (SC/ST, OBC, others), Children ever born (1, 2, 3, 4+), Place of delivery (public, private) were included based on past literature^19–22^.

### Analytical approach

The analysis was carried out using the statistical software STATA (Version 16), and the maps were generated with the help of spatial analysis software ArcGIS. We began with descriptive statistics to understand the frequency distributions of the study variables across States and Union Territories (UTs) of the NFHS-5 survey. Bivariate analyses were conducted to measure the prevalence of unmet needs for family planning by selected predictor variables classified at state levels. Subsequently, variation in contraceptive demand satisfied with modern contraceptive methods across different states over two rounds of NFHS (4&5) has been analyzed by some selected background characteristics of married women aged 15–49. Further, quintile regression analysis was adopted to understand the coefficients for the total unmet need for family planning and unmet need for spacing by selected women's characteristics. Quintile regression analysis is an essential technique, unlike simple linear regression, which uses the least-squares method to estimate the conditional mean of the variables. Quantile regression can be explained as the extension of linear regression used when the conditions of linear regression cannot explain the regression model^23^. The reason behind conducting quantile regression was that the dependent variables were not fulfilling the linearity conditions along with normality, homoscedasticity, and test of independence. In this situation, quantile regression was the most appropriate technique to estimate the median values at different percentiles. Quantile regression result depicts the significant association between independent variables with dependent variables’ total unmet need for family planning and an unmet need for spacing at different quantiles (q25, q50, q75, q90).

**Figure 1:**
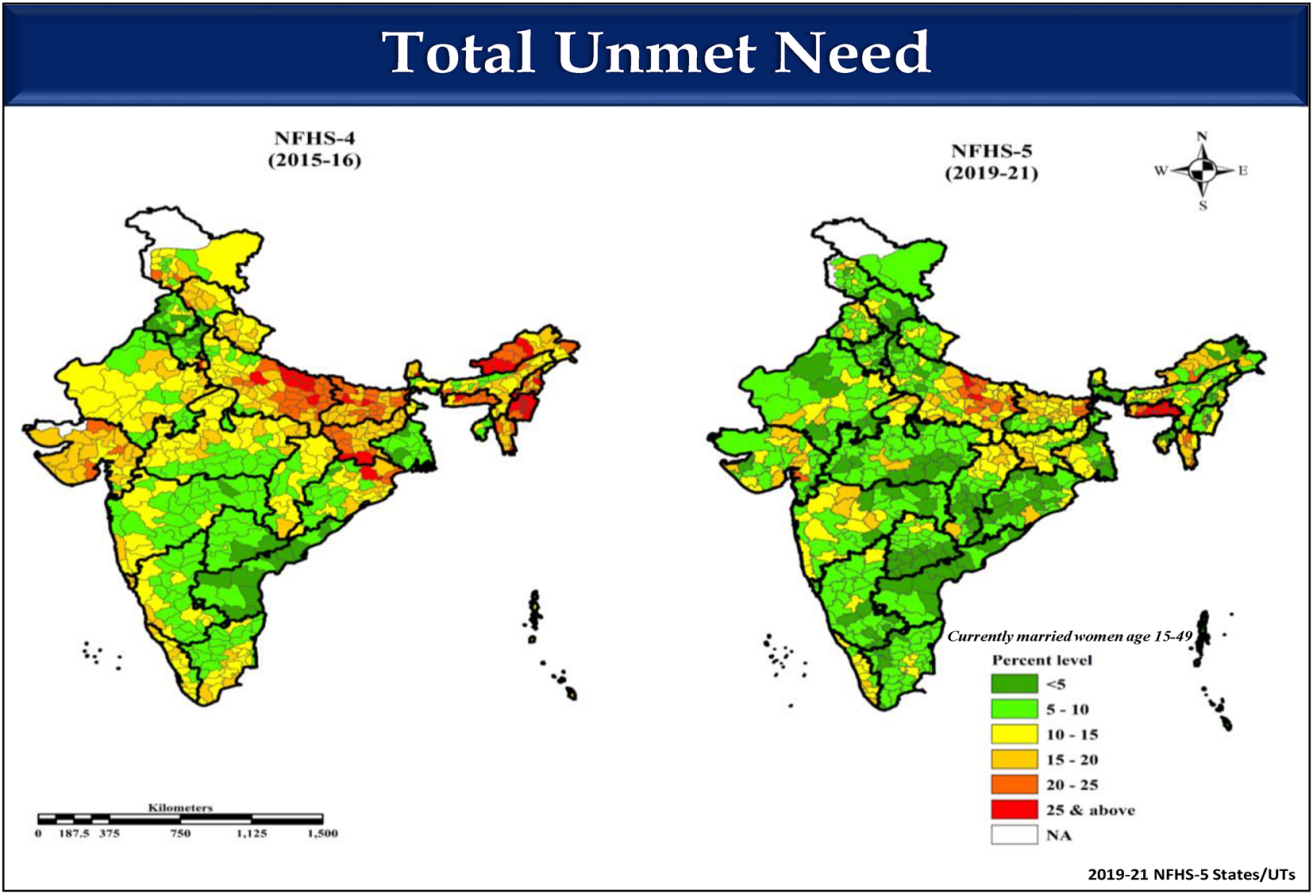
Percent distribution of currently married women aged 15–49 having unmet need for family planning in India, NFHS-4 (2015–2016) and NFHS-5 (2019–2021).

Lastly, a multilevel logistic regression analysis (three-level: District, PSU, and Household) was conducted using a set of contextual variables such as residence, religion, caste, wealth index, place of delivery, and individual-level variables like age, schooling, and children ever born within a hierarchal framework. We have employed multilevel logistic regression to understand the variation and key drivers of unmet need of family planning adopting hierarchal framework, where women are nested in household, household are nested in PSUs, and PSUs are nested in a district. It provides us extent of clustering in variable under study at different levels. In a preliminary analysis of multilevel modelling a baseline or an intercept model was examined only to assess the extent of the dependent variable’s variation between HHs, between PSUs and between districts. Subsequently, we have included a set of contextual and individual characteristics added to the baseline model to decode the extent of clustering at different levels. Appropriate sampling weight was utilized in the entire analysis to expand the representation of the samples included in the analysis. Lastly, the choropleth map (Fig. 1) was generated with the help of spatial analysis software ArcGIS (Version 10.5) (https://www.arcgis.com/index.html).

### Ethical approval and consent to participate

The present analysis utilizes a secondary data set with no identifiable information on the survey participants. This dataset is available in the public domain for research use; hence no approval was required from any institutional review board as there is no question of human subject protection in this case.

## Results

### Has unmet need declined nationally?

The prevalence of unmet need in India declined nationally from 12.9% in NFHS-4 to 9.4% in NFHS-5.

### Is there regional variation in unmet need?

Variation in the prevalence of total unmet need for family planning from NFHS-4 to NFHS-5 is presented in Table 1. The north-eastern states showed a significant decline in the prevalence of unmet needs for family planning in Manipur (17.8% point), Nagaland (13.5%), and followed by Sikkim (9.1% point). In the northern region, the highest decrease in the prevalence of total unmet needs was reflected in Delhi (8.9%), and it was the lowest in Punjab (3.7%). However, the central region states documented a slight variation (3–5%) in the prevalence of unmet needs for family planning. Results from the Eastern states showed a substantial decline in Bihar (8.7%).

**Table 1:** Variation in total unmet need for family planning among currently married women aged 15–49 in India, NFHS (2015–2021).

States	Total unmet need			
	NFHS-4 (2015–2016)	(95% CI)	NFHS-5 (2019–2021)	(95% CI)
North				
Delhi	15.0	14.6–15.8	6.1	5.4–6.8
Himachal Pradesh	15.1	14.4–17.1	7.6	7.0–8.1
Jammu & Kashmir	13.0	11.6–13.1	8.1	7.2–8.4
Punjab	6.2	5.6–6.8	9.9	9.2–10.6
Rajasthan	12.0	11.8–2.8	7.6	7.2–8.0
Uttarakhand	16.0	14.5–16.6	8.8	8.0–9.7
Central				
Chhattisgarh	11.0	10.5–11.7	8.3	7.7–8.8
Madhya Pradesh	12.0	12.7–14.3	7.7	7.3–8.1
Uttar Pradesh	18.0	17.7–18.4	12.8	12.5–13.2
East				
Bihar	21.1	20.6–21.7	12.8	13.0–14.1
Jharkhand	18.0	17.7–19.1	11.5	10.9–12.1
Odisha	14.0	13.0–14.3	7.2	6.6–7.7
West Bengal	8.1	6.8–8.1	7.3	6.5–7.6
Northeast				
Arunachal Pradesh	22.0	20.5–22.7	12.4	11.5–13.4
Assam	13.6	13.4–14.9	10.5	10.4–11.5
Manipur	29.6	28.7–31.4	11.8	10.9–13.6
Meghalaya	21.2	19.8–22.6	26.1	25.3–28.6
Mizoram	20.8	18.3–21.5	17.8	17.1–20.6
Nagaland	21.6	20.8–23.5	8.1	7.81.05
Sikkim	20.3	19.7–23.7	11.2	8.6–15.3
Tripura	8.9	9.1–12.3	8.5	7.3–9.1
West				
Goa	17.4	14.6–20.4	8.4	5.5–11.2
Gujarat	15.7	16.1–17.8	10.4	9.8–10.9
Maharashtra	9.2	9.1–10.3	9.0	8.7–10.4
South				
Andhra Pradesh	4.7	4.1–5.2	4.8	4.2–5.2
Karnataka	9.9	9.7–11.1	6.5	5.4–6.4
Kerala	13.3	12.7–14.7	12.1	11.5–13.4
Tamil Nadu	10.0	6.5–8.1	7.5	6.9–8.0
Telangana	7.4	6.5–8.1	6.6	6.0–6.9
India	12.9	12.7–13.0	9.4	9.3–9.6

In comparison, a negligible decline (0.8%) in the prevalence of unmet need for family planning was noted in West Bengal. However, the southern states reported a slight change (decline) in the prevalence of unmet need for family planning. The states such as Maharashtra, Tripura, and Andhra Pradesh have yet to report any change in the prevalence of total unmet needs for family planning.

### Is there a demographic variation in unmet need based on maternal age, education, religion, caste, wealth index, family size, and access to antenatal care (ANC)?

The magnitude and intensity of the coefficients for the total unmet need for family planning and unmet need for spacing are presented in Table 2. We have utilized the quintile regression to estimate the coefficients of the total unmet need for family planning and unmet need for the spacing model at 25%, 50%, 75%, and 90% quantiles. There is a significant association between years of schooling with total unmet need for family planning at (q25) quantiles and an unmet need for spacing at (q25, q50) quantiles. Notably, female literacy has positive and statistically significant associations with the total unmet need for family planning at q75 and q90 quantiles.

**Table 2:** Coefficient of quantile regression of total unmet need and unmet need for spacing by some selected characteristics among currently married women aged 15–49 in India (NFHS-5, 2019–21).

Characteristics	Total unmet need for family planning							
Quartile	q25	P-value	q50	P-value	q75	P-value	q90	P-value
Sanitation facility	**− 0.07**	**(p < 0.001)**	**− 0.06**	**(p < 0.001)**	− 0.03	(p < 0.289)	− 0.05	(p < 0.141)
Schooling	**0.07**	**(p < 0.001)**	0.08	(p < 0.05)	− 0.03	(p < 0.486)	− 0.08	(p < 0.051)
Child marriage	0.02	(p < 0.325)	0.02	(p < 0.512)	− 0.04	(p < 0.124)	− 0.12	(p < 0.001)
Birth order	− 0.18	(p < 0.254)	− 0.26	(p < 0.067)	0.03	(p < 0.920)	0.47	(p < 0.106)
FP worker talked to a non-user	− 0.02	(p < 0.311)	− 0.02	(p < 0.413)	− 0.06	(p < 0.067)	− 0.05	(p < 0.156)
ANC visits	**− 0.06**	**(p < 0.001)**	**− 0.10**	**(p < 0.001)**	**− 0.13**	**(p < 0.001)**	**− 0.12**	**(p < 0.001)**
Skilled birth attendant	− 0.03	(p < 0.432)	− 0.03	(p < 0.393)	− 0.08	(p < 0.240)	**− 0.18**	**(p < 0.001)**
Place of residence	**− 0.03**	**(p < 0.001)**	**− 0.03**	**(p < 0.001)**	− 0.03	(p < 0.05)	**− 0.05**	**(p < 0.005)**
Caste	− 0.03	(p < 0.068)	0.00	(p < 0.997)	0.00	(p < 0.962)	− 0.02	(p < 0.156)

Quartile	Unmet need for spacing							
	q25	P-value	q50	P-value	q75	P-value	q90	P-value
Sanitation facility	**− 0.02**	**(p < 0.001)**	0.00	(p < 0.538)	0.02	(p < 0.259)	**0.06**	**(p < 0.005)**
Schooling	**0.03**	**(p < 0.001)**	**0.03**	**(p < 0.001)**	− 0.01	(p < 0.605)	− 0.03	(p < 0.057)
Child marriage	**0.02**	**(p < 0.05)**	0.02	(p < 0.087)	0.00	(p < 0.728)	− 0.02	(p < 0.274)
Birth order	− 0.05	(p < 0.505)	− 0.05	(p < 0.636)	0.09	(p < 0.615)	**0.54**	**(p < 0.005)**
FP worker talked to a non-user	0.01	(p < 0.560)	0.00	(p < 0.907)	− 0.02	(p < 0.05)	**− 0.04**	**(p < 0.05)**
ANC visits	**− 0.03**	**(p < 0.001)**	**− 0.03**	**(p < 0.001)**	**− 0.04**	**(p < 0.001)**	− 0.01	(p < 0.341)
Skilled birth attendant	− 0.01	(p < 0.636)	− 0.02	(p < 0.075)	− 0.04	(p < 0.222)	**− 0.14**	**(p < 0.001)**
Place of residence	**− 0.01**	**(p < 0.05)**	**− 0.02**	**(p < 0.001)**	− 0.01	(p < 0.283)	− 0.02	(p < 0.05)
Caste	0.00	(p < 0.539)	− 0.01	(p < 0.281)	0.02	(p < 0.05)	**0.04**	**(p < 0.001)**

Significant values are in bold.

Living in an urban area was associated with the unmet need for family planning and the unmet need for spacing across the quantiles. Furthermore, women belonging to Scheduled Caste (SC) and Scheduled Tribe (ST) communities have significant associations with the unmet need for spacing at q90 quantiles. ANC visits are associated with the unmet need for family planning and the unmet need for spacing across the quantiles. Figure 1 presents the spatial distribution of the percent of total unmet need for family planning in Indian states for NFHS-4 and NFHS-5. It is evident from the spatial map that the red areas depicting hotspot regions (25 & above unmet need) have decreased from NFHS-4 to NFHS-5.

Table 3 presents the unmet need for family planning, and the demand satisfied for family planning by the selected background characteristics. The unmet need for family planning was 9.4 percent, while the unmet need for spacing and the unmet need for limiting was 4% and 5.4%, respectively. Notably, the percentage of demand satisfied was 87.9 percent. Results depict that the demand for the unmet need for spacing and the unmet need for limiting was the highest among the women in the age categories 15–19 and 20–24. However, the demand was least satisfied (61.3%) in the 15–19 age group. In addition, demand satisfaction improved as the age of the women increased. Women living in rural areas had higher demand for unmet need for spacing (3.5%) and unmet need for limiting (4.9%) than women living in urban localities. The demand for unmet need for spacing and unmet need for limiting increased as educational attainment increased, the demand for unmet need for spacing (2.0%) and unmet need for limiting (5.3%) was the lowest among illiterate women, and the demand was highest among the women who had studied for 12 or more years. The demand for limiting was the highest (6.8%) among Muslim women. Demand for spacing was the highest (5.5%) among Christian women; there was not much variation in demand satisfaction across the different religions. Along the same line, slight variation in demand of unmet need for spacing and limiting was documented throughout the caste categories. Across wealth quantile categories, the overall unmet demand (11.4%) for spacing and limiting was the highest among the women belonging to the lower strata of society.

**Table 3:** Percentage of currently married women aged 15–49 with unmet need for family planning, and percentage of the demand for family planning satisfied, by background characteristics, India, NFHS, 2019–21.

Background characteristic	Unmet need for family planning					
	For spacing	For limiting	Total	Percentage of demand satisfied	p values	Number of women
			Unmet need			
Age					p < 0.001	
15–19	15.6	2.2	17.8	61.3		15,407
20–24	12.4	4.9	17.3	71.1		71,584
25–29	6.2	7	13.2	82.2		1,02,257
30–34	2.5	6.6	9.1	89		93,946
35–39	0.9	5.5	6.3	92.6		90,684
40–44	0.3	4.7	5	93.9		73,706
45–49	0.2	3.3	3.4	95.5		73,768
Place of residence					p < 0.001	
Urban	3.5	4.9	8.4	89.2		1,63,394
Rural	4.3	5.6	9.9	86.9		3,57,957
Schooling					p < 0.001	
No schooling	2	5.3	7.3	90.6		1,43,754
< 5 years complete	2.4	4.9	7.2	90.9		32,023
5–7 years complete	2.9	5.4	8.3	89.4		79,079
8–9 years complete	4.7	5.5	10.3	86.5		85,112
10–11 years complete	4.8	5.1	9.9	86.7		66,173
12 or more years complete	6.9	5.6	12.5	82.7		1,15,211
Religion					p < 0.001	
Hindu	3.9	5.2	9	88.3		4,27,114
Muslim	5	6.8	11.8	83.6		68,631
Christian	5.5	4.8	10.4	85.6		11,391
Sikh	3.5	6	9.5	87.7		8,080
Buddhist/Neo-Buddhist	3.9	4.3	8.2	89.1		3,081
Jain	4.8	2.7	7.5	90.8		1,173
Other	4.5	6.1	10.7	84.9		1,883
Caste/tribe					p < 0.001	
Scheduled caste	4	5.2	9.2	87.9		1,12,610
Scheduled tribe	4.5	4.7	9.2	87.4		47,852
Other backward class	4.2	5.4	9.6	87.4		2,24,682
Other	3.7	5.7	9.4	87.9		1,32,179
Don't know	5	6.9	12	83.4		4,029
Wealth quintile					p < 0.001	
Lowest	4.5	6.9	11.4	84.6		97,962
Second	4.1	5.6	9.7	87.2		1,04,135
Middle	3.9	4.8	8.6	88.7		1,06,487
Fourth	4	5	9	88.3		1,08,247
Highest	3.7	4.8	8.6	89		1,04,520
Total	4	5.4	9.4	87.6		5,21,352

Table 4 presents the results of the multilevel logistic regression model, which was used to examine the key drivers of total unmet needs for family planning adopting a hierarchal model focusing on fixed and random effects. The intraclass correlation coefficient in the mixed model (Random effect and fixed effect model) was the highest at the household level (30%) followed by PSU (19%), and district level (10%), a considerable improvement in comparison to the null model. The corresponding ICC value in the null model were 18, 16, and 8 percent. This clustering in unmet need for family planning at different levels is consistent with the estimated variance in unmet need for family planning at district (47%), PSU (43%), and households (51%) levels.

**Table 4:** Random effect and fixed effect model showing critical drivers of total unmet need in Family Planning in India, NFHS 2019–21.

Total unmet need	Null model (95% CI)	Model I with an adjusted odds ratio (95% CI)		
Age				
15–29®				
30–39		1.01	p = 0.702	(0.96–1.06)
40–49		1.33***	p < 0.001	(1.18–1.50)
Schooling				
No schooling®				
Up to five		1.04	p = 0.807	(0.96–1.12)
Five to 10		0.98	p = 0.871	(0.91–1.05)
Ten and above		1.04	p = 0.040	(0.97–1.13)
Place of residence				
Urban®				
Rural		0.98	p = 0.849	(0.92–1.05)
Religion				
Hindu®				
Muslim		1.17***	p < 0.001	(1.09–1.26)
Others		1.07	p = 0.065	(0.96–1.19)
Caste				
SC/ST®				
OBC		1	p = 0.336	(0.95–1.07)
Others		1.02	p = 0.072	(0.95–1.09)
Children ever born				
1®				
2		0.81***	p < 0.001	(0.77–0.86)
3		0.73***	p < 0.001	(0.68–0.78)
4+		0.83***	p < 0.001	(0.77–0.90)
Wealth Index				
Poorest®				
Poorer		1.02	p = 0.148	(0.95–1.08)
Middle		1.04	p = 0.407	(0.97–1.11)
Richer		1.14***	p < 0.001	(1.06–1.23)
Richest		1.01	p = 0.382	(0.92–1.10)
Place of delivery				
Public®				
Private		0.97	p = 0.368	(0.89–1.05)
Random effects				
Variance				
District	0.33 (0.29–0.37)	0.47		(0.39–0.57)
PSU	0.32 (0.30–0.34)	0.43		(0.38–0.49)
Household	0.32 (0.04–0.20)	0.51		(0.30–0.88)
ICC values				
District	0.08 (0.07–0.09)	*0.1*		(0.86–0.12)
PSU	0.16 (0.15–0.17)	*0.19*		(0.18–0.21)
Household	0.18 (0.17–0.20)	*0.3*		(0.25–0.36)

*ICC* intra class coefficients, *PSU* primary sampling unit, (***p < 0.001).

Significant values are in italics.

Decoding the factors responsible for the total unmet need for family planning using a fixed effect model indicate significantly higher odds among women aged 40–49 (AOR = 1.33, 95% CI 1.18–1.50) than those aged 15–29. The odds were higher for Muslim women (AOR = 1.17, 95% CI 1.09–1.26) for total unmet family planning needs than women belonging to the Hindu religion. The predictor children ever born showed a statistically significant association with total unmet need for family planning among women with 2, 3, and 4+ children compared to women with a single child. Results highlighted the significant positive association with economic status; women from the richer wealth quintile (AOR = 1.14***, 95% CI 1.06–1.23) were more likely to have a higher unmet need for family planning than the women belonging to the poorest wealth quintile.

## Discussion

This study aims to analyse the changes in the prevalence of unmet need for family planning among married women aged 15–49, to document existing variability, and to identify key drivers within a hierarchal framework. We employed quintile regression and multilevel modelling to decode factors responsible for clustering in the total unmet need of family planning at different levels.

Our results reveal that total unmet need for limiting and spacing has declined nationally. We found a significant reduction in the prevalence of unmet need for family planning and unmet need for spacing in the last 6 years, from NFHS-4 to NFHS-5. These findings are consistent with the downward trend observed in previous national surveys regarding unmet need for family planning and spacing^8,24^.

We documented substantial regional variation in unmet need for family planning. In this context, the sharpest decrease in the prevalence of unmet need for family planning was in Manipur (17.8% point), Nagaland (13.5%), and followed by Sikkim (9.1%). The Family planning programme (incentive-based program) focusing on spacing at births was implemented in 18 states including EAG, Northeastern, Gujarat, and Haryana, which may help explain the decline in the unmet need for spacing in Northeastern states. The results from the multilevel analysis reveal a substantial increase in clustering in unmet need of family planning at the household level in Model I (30%) as compared to the null model (18%) indicate the importance of segregating household-level interventions to eliminate the burden of unmet need in family planning in India.

Our data demonstrated that there is a demographic variation in unmet need based on maternal age, education, religion, caste, wealth index, family size, and access to antenatal care (ANC). Our results reveal that multiple demographic variables impact the unmet family planning needs. The mother's age, education, place of residence, religion, and caste were some of the most prominent predictors of the unmet need for family planning. Our results reveal a significant association between years of schooling with the total unmet need for family planning at (q25) quantiles and an unmet need for spacing at (q25, q50) quantiles. Our study found that the demand for the unmet need for spacing and limiting was the highest among women aged 15–19 and 20–24. Several previous studies have documented the contrary to this finding showing evidence between age at marriage and current age with the unmet need for family planning^25–29^. They revealed that the odds of a total unmet need for family planning were higher among women aged 40–49 (AOR = 1.33*) than those aged 15–29. Further, women belonging to SC/ST community have significant associations with the unmet need for spacing at q90 quantiles. Findings from the quintile regression indicate that female literacy has positive and statistically significant associations with the unmet need for family planning at q75 and q90 quantiles. Our data are consistent with a study on tribal women revealing that women's age and education have significant associations between modern contraception and the unmet need for family planning^30^. The demand for limiting was the highest (6.8%) among Muslim women, and the odds were higher for Muslim women (AOR = 1.17) for total unmet need for family planning compared to women belonging to the Hindu religion. In our study, the predictor children ever born show a statistically significant association with a total unmet need for family planning among women with 2, 3, and 4+ children compared to women with a single child^31^.

We found that indicators of ANC visits have statistically significant associations with the unmet need for family planning and the unmet need for spacing across the quantiles. There is a lag between knowledge and the use of contraceptive methods in developing countries like India; the perception of side effects may be a reason for this problem. Previous studies have also elaborated the fact that currently married women who reported 'high discussion' on family planning compared to those with 'low discussion' were seven times more likely to use modern contraceptives for birth spacing^32^. The national fact sheet (NFHS-4) reported that only 19 percent of female non-users talked to a health worker about family planning, and 50 percent of current users never talked about the side effect of the existing method. Through the National Rural Health Mission (NRHM), India is currently experiencing a substantial improvement in the health sector, particularly with regard to maternal health. This highlights the significance of the front line workers having conversations about family planning and engaging with women about contraception and other important issues of reproductive health.

## Strengths and limitations of the study

The strengths of this study on changing discourse of the unmet need for family planning are rooted in the analysis of sociocultural and ecological drivers of behavior. We adopted a hierarchal framework in which women are nested in households, household are nested in PSUs, and PSUs are nested in a districts. As a result, identification of key drivers at different levels (household, PSU and districts) provides unique insights into the kinds of multi-faceted interventions that could further change discourses of unmet need of family planning as a key marker of reproductive health programs in the country. These findings will enable the government to devise strategies having hyperlocal solutions to address the unmet need of family planning. One limitation of this study was restricting the sample to only women who were currently in a union at the time of the survey. Around one-fourth of women aged 15–49 were not currently married at the time of the survey in NFHS-4 and NFHS-5. However, many of such women might be sexually active and may experience unmet needs for family planning. Another limitation of the study is the lack of measurement of causality between outcome and independent variables using cross-sectional data.

## Future directions

In view of the changing landscape of family planning practices especially among young couples in India, the unmet need for contraceptive usage for spacing and delaying the first pregnancy calls for more granular understanding beyond general segmentation of users by broader socio-economic categories. Future studies should include focus on social and gender norms using the lens of intra-household power dynamics. The studies should try to capture insights not just from the wives but also include mothers-in-law, and husbands. If possible, some insights from the community health workers and local influencers would provide novel insights into how to design human centered programs where the focus shifts from emphasizing contraceptive methods to user experience.

## Conclusion

The study findings indicate a considerable decline in the unmet need for family planning and the unmet need for spacing during the period 2016–21 (NFHS-4 to 5). The study concludes that the predictors like years of schooling, place of residence, caste, religion, wealth quintile, number of ANC visits, mother’s age and children ever born are significantly associated with the total unmet need for family planning and the unmet need for spacing in most of the districts in India. However, the predictors and its corresponding effect on influencing unmet needs for contraception is not uniform for women of different quantiles. The ongoing family planning program should use the study findings to come up with better segmentation of women with unmet need and accordingly design solutions to convert them into active users of modern contraception. The program should strive to shift its focus from widening the basket of choice to user centric approach with granular understanding of various determinants and their relative strength on influencing the family planning behavior of each category of women. We conclude that greater access to frontline health workers among young wives, more significant investment in education in general, more reproductive health education for females in particular, as well as harnessing greater technological access will continue to reduce the unmet needs for family planning.

### Supplementary Information

Supplementary Tables.

## Data Availability

The dataset analyzed in the current study is available online on the official website of the Demographic Health Survey Program https://www.dhsprogram.com. However, the datasets used can be made available from the corresponding author upon reasonable request.
