# Supplementary material for: Changes in discourse on unmet need for family planning among married women in India: evidence from NFHS-5 (2019–2021)
Source: Sci Rep. 2023 Nov 22;13:20464. doi: 10.1038/s41598-023-47191-9 (PMC10665352; doi:10.1038/s41598-023-47191-9)
Supplement: Supplementary file 1 — Supplementary Tables. [file 41598_2023_47191_MOESM1_ESM.docx]

| **Table S1: Supplementary table showing variance inflation factor (VIF) as test of multicollinearity among predictors included in the model** | | |
| --- | --- | --- |
| **Age** | **Variance Inflation factor (VIF)** | **1/VIF** |
| **15-29 ®** |  |  |
| 30-39 | 1.69 | 0.593 |
| 40-49 | 1.10 | 0.907 |
| **Schooling** | |  |
| **No schooling ®** | |  |
| Up to five | 1.48 | 0.675 |
| Five to 10 | 3.08 | 0.325 |
| Ten and above | 3.47 | 0.288 |
| **Residence** | |  |
| **Urban ®** |  |  |
| Rural | 3.60 | 0.278 |
| **Religion** |  |  |
| **Hindu ®** |  |  |
| Muslim | 1.24 | 0.807 |
| others | 1.25 | 0.803 |
| **Caste** |  |  |
| **SC/ST ®** |  |  |
| OBC | 2.32 | 0.431 |
| Others | 1.76 | 0.568 |
| **Children Ever born** |  |  |
| **1 ®** |  |  |
| 2 | 1.93 | 0.518 |
| 3 | 1.50 | 0.665 |
| 4+ | 1.62 | 0.617 |
| **Wealth Index** |  |  |
| **Poorest ®** |  |  |
| Poorer | 1.93 | 0.506 |
| Middle | 2.07 | 0.482 |
| Richer | 2.27 | 0.441 |
| Richest | 2.59 | 0.386 |
| **Place of delivery** |  |  |
| Public ® |  |  |
| Private | 1.57 | 0.635 |
| **Mean VIF** | **2.03** |  |

| **Table S2: Decision tree depicting the selection process of women aged 15-49 having unmet need, NFHS-4 & 5** | |
| --- | --- |
| **Total number of Households (NFHS-4)**  601, 509 | **Total number of Households (NFHS-5)**  636, 699 |
|  |  |
|  |  |
| **Total women aged 15-49 (NFHS-4)**  699,686 | **Total women aged 15-49 (NFHS-5)**  724,115 |
|  |  |
|  |  |
| **Currently married women aged 15-49 (NFHS-4)**  499,627 | **Currently married women aged 15-49 (NFHS-5)**  512,408 |
|  |  |
|  |  |
| **Currently married women aged 15-49 using any method of contraception (NFHS-4)**  404,635 | **Currently married women aged 15-49 using any method of contraception (NFHS-5)**  353, 816 |
| **Sterilised and pregnant women aged 15-49 (NFHS-4)**  215,101 | **Sterilised and pregnant women aged 15-49 (NFHS-5)**  206,405 |
|  |  |
| **Sexually active currently married women aged 15-49 having unmet need (NFHS-4)** | **Sexually active currently married women aged 15-49 having unmet need (NFHS-5)** |
| 71, 531 (**Unweighted**)  68,626 (**Weighted**) | 51,624 (**Unweighted**)  48,855 (**Weighted**) |
